# Biocontrol of *Rhizoctonia solani* in basmati rice by the application of *Lactobacillus* and *Weissella* spp*.*

**DOI:** 10.1038/s41598-023-41058-9

**Published:** 2023-08-24

**Authors:** Maira Akhtar, Asia Nosheen, Rumana Keyani, Humaira Yasmin, Rabia Naz, Saqib Mumtaz, Muhammad Nadeem Hassan

**Affiliations:** Department of Biosciences, COMSATS University Islamabad, Park Road, Chak Shahzad, Islamabad, 44000 Pakistan

**Keywords:** Plant physiology, Plant stress responses

## Abstract

Rice is a staple food crop and is a major source of employment and income in the world. However, the attack of fungal disease poses a serious threat to the crop growth and productivity and leads toward yield loses. Therefore, current study was performed to evaluate the biocontrol potential of *Lactobacillus* and *Weissella* spp. on basmati rice against *Rhizoctonia solani.* Agar disc method was performed to evaluate the antifungal activity of both bacteria against *R. solani.* Petri plate and pot experiments were conducted to evaluate the growth promotion and biocontrol potential of both bacteria in Basmati rice under *R. solani* stress*.* Results indicated that maximum antifungal activity (82%) was recorded by *Lactobacillus sp*. Maximum phosphate solubilization and siderophore production was recorded by *Weissella* sp. In petri plate experiment, maximum root length, root fresh and dry weight (36%, 40% and 13%) was recorded by *Weissella* sp. and maximum shoot length and shoot fresh weight (99% and 107%) by *Lactobacillus sp*. In pot experiment, both bacteria enhanced the growth parameters of Basmati rice including root and shoot length, fresh and dry weight as well as no. of lateral roots. Application of *Weissella* sp. resulted in maximum increase (332% and 134%) in chlorophyll a and b content while *Lactobacillus* sp. + *R. solani* showed maximum (42%) carotenoid contents. *Lactobacillus* sp. + *R. solani* showed maximum increase in the proline (54%) and sugar contents (100%) while *Lactobacillus* sp. alone showed maximum (35%) soluble protein contents. Plant defense enzymes i-e SOD (400%), POD (25%), CAT (650%), PPO (14%) and PAL (124%) were notably increased by *Weissella* sp. + *R. solani* and *Lactobacillus sp* + *R. solani.* The *Lactobacillus sp* showed the best results in antifungal activity against *R. solani* and *Weissella* sp. showed the best results in production of defense enzymes in basmati rice against *R. solani* stress and can be suggested as the potent biocontrol agents for the rice crop.

## Introduction

Rice is the main staple food and economic crop for almost half of the world population^1^. It is a part of daily calorie intake of more than 3 billion people around the globe and India, China, Bangladesh, Pakistan are the major rice consumers^2^. Rice is grown over an area of 2.7 million ha in 2016–2017 in Pakistan with 7 million tons of rice production annually^3^. Basmati rice of Pakistan is famous globally due to its specific aroma^4^. But the yield of Basmati rice is continuously decreasing due to high disease incidence^5^. Pathogenic fungi in plants cause various infection in all the developmental stages of fruits and vegetable, in rice, soil borne fungi are the most damaging^6^.

Sheath blight is a disease of rice caused by the fungal pathogen *Rhizoctonia* (*R. solani*) and causes devastating loss in rice growth and yield^7^. Farmers deployed a number of protection strategies to the rice field in order to protect the crop from diseases^8^. However, excessive use of chemical pesticides is toxic to the environment and human beings^9^. Therefore, it is the need of an hour to find substitutes of these synthetic chemicals with the natural ones. Use of beneficial bacteria as a biocontrol agent can be a better alternative^10^.

Beneficial bacteria as a biocontrol agent are highly specific and effective in order to control crop diseases^11^. These microorganisms are reported to release chemicals that disrupt the cellulose, protein, hemicelluloses or DNA of the pathogen^12^. Bacterial biocontrol agent is widely used to control fungal diseases in plants which even in minor amount in soil result in whole plant destruction. The molecules such as hydrogen cyanide, ammonia and antibiotics such as pyrrolnitrin, pyolutrin and 2,4-diacetylphloroglucinol are known to suppress variety of fungal diseases^6^. These biocontrol agents have a variety of advantages (1) improve plant growth (2) release hormones that play a key role in defense responses against the pathogen (3) release secondary metabolites that help to control crop diseases. Some of the bioactive compounds of bacteria show antifungal and antimicrobial properties which make them a potent biocontrol agent^13^.

Nowadays, probiotics are in high demand due to its various antimicrobial and antifungal properties. Probiotics are bacteria that provide great benefits to the host mostly include protection against the pathogen invasion and increase nutritional viability in host. They also provide protection against the pathogen that attack humans and cause diseases^14^. Lactic acid bacteria, some yeast and molds are the most common in probiotics and also used as biocontrol agent^15^.

The aim of the current study was to evaluate the biocontrol potential of *Lactobacillus* and *Weissella* spp. against *R. solani* in Basmati rice. In order to accomplish the aim of the study, antagonist assay was performed and then for the evaluation of the growth promotion effect and biocontrol potential of *Lactobacillus* sp. and *Weissella* sp. on basmati rice, a pot experiment was carried out. Similarly, changes in antioxidant and enzymatic activity of basmati rice under *R. solani* attack were recorded.

## Results

### Antagonist assay

To evaluate the antifungal potential of *Lactobacillus* sp. (accession no. OR100385) and *Weissella* sp. (accession no. OR100388) against *R. solani,* an antagonist assay was performed (Supplementary Fig. 1A). Both biocontrol agents resulted in the inhibition of growth of pathogenic fungus (*R. solani*). However, *Lactobacillus* sp. showed maximum (81%) inhibition of the pathogenic fungus and *Weissella* sp. resulted in 60% inhibition of *R. solani* as compared to the control fungus (Table 1).

**Table 1:** In vitro antifungal activity of *Lactobacillus* sp. and *Weissella* sp. against *Rhizoctonia solani.*

Bacterial Strains Treatment	*Rhizoctonia solani* growth	
	Mycelial Growth (cm)	Percentage Inhibition %
*Lactobacillus* sp.	1.63 ± 0.06^b^	81.8
*Weissella* sp.	3.6 ± 0^a^	60

Data are expressed as mean ± standard deviation of three replicates.

All means which are not sharing letter are significantly different at *P* < 0.05 while those are sharing are non-significant.

### Detection of phosphate solubilization, siderophore and hydrogen cyanide production potential of *Lactobacillus* sp. and *Weissella* sp.

Phosphate solubilization assay of *Lactobacillus* sp. and *Weissella* sp. was performed to access the solubilization index of phosphate. Both the bacterial isolates have shown a potential to solubilize phosphate which is shown in the form of formation of halo zones in Supplementary Fig. 1B. However, *Weissella* sp. showed more potential to solubilize phosphate as compared to *Lactobacillus* sp. (Table 2)*.* Formation of halo zones indicate the siderophore production potential of bacterial strains in the given media (Supplementary Fig. 2A). In the current study, compared to *Lactobacillus* sp.*, Weissella* sp. showed more ability to produce siderophore (Table 2). The results of hydrogen cyanide (HCN) production by the bacterial strains are shown in Supplementary Fig. 2B. No HCN production was observed by *Lactobacillus* sp. However, *Weissella* sp. showed very weak ability for the HCN production which is shown as orange color in the Fig. 2B (Table 2).

**Table 2:** Evaluation of *Lactobacillus* and *Weissella* spp. for phosphate solubilization, siderophore and HCN production.

Bacterial Strains	Phosphate Solubilization Index	Siderophore Production	HCN Production
*Lactobacillus* sp.	2.13 ± 0.005^b^	0.6 ± 0^b^	−
*Weissella* sp.	4.45 ± 0.65^a^	1.46 ± 0.03^a^	+

(+) stands for weak producer and (−) stand for not producer.

Data are expressed as mean ± standard deviation of three replicates.

All means which are not sharing letter are significantly different at *P* < 0.05 while those are sharing are non-significant.

### Rate of seed germination

Germination data (germination percentage, radical length and plumule length) was recorded during the first four days of petri plate experiment (Table 3). The germination percentage of *Lactobacillus* sp. treatment at the fourth day showed remarkable increase of 100% as compared to control. *Weissella* sp. and the control showed 93% increase in the rate of seed germination. Plumule and radical length of *Weissella* sp. and *Lactobacillus* sp. also showed increase as compared to the control. Radical length of the rice seedlings treated with *Lactobacillus* and *Weissella* spp. showed increase of 34% and 53% as compared to the control respectively. The *Lactobacillus sp.* showed maximum increase (7%) in plumule length as compared to the control and *Weissella* sp. at the fourth day of the experiment (Table 3).

**Table 3:** Effect of *Lactobacillus* and *Weissella* spp. on the rate of seed germination, radical and plumule length of rice.

Day	Treatments	Rate of seed germination (%)	Radical Length (cm)	Plumule Length (cm)
1	Control	0 ± 0	0 ± 0	0 ± 0
	*Weissella* sp.	0 ± 0	0 ± 0	0 ± 0
	*Lactobacillus* sp.	0 ± 0	0 ± 0	0 ± 0
2	Control	26.6 ± 6.66^a^	0.1667 ± 0.03^a^	0.133 ± 0.03^a^
	*Weissella* sp.	26.6 ± 6.66^a^	0.2 ± 0.03^a^	0.1 ± 0.16^a^
	*Lactobacillus* sp.	33.3 ± 6.66^a^	0.233 ± 00^a^	0.1667 ± 0.057^a^
3	Control	80 ± 0^a^	0.5 ± 0.05^a^	0.5 ± 0^a^
	*Weissella* sp.	53.3 ± 6.66^c^	0.433 ± 0.033^a^	0.5 ± 0.15^a^
	*Lactobacillus* sp.	73.3 ± 7.6^b^	0.5667 ± 0.088^a^	0.5 ± 0.05^a^
4	Control	93.33 ± 6.66^a^	0.8667 ± 0.03^a^	1.5 ± 0^a^
	*Weissella* sp.	93.33 ± 0^a^	1.33 ± 0.088^a^	1.4 ± 0.15^a^
	*Lactobacillus* sp.	100 ± 3.33^a^	1.1667 ± 0.120^a^	1.6 ± 0.17^a^

Data are expressed as mean ± standard deviation of three replicates.

All means which are not sharing letter are significantly different at *P* < 0.05 while those are sharing are non-significant.

### Growth parameters in petri plate experiment

The results of petri plate experiments showed that maximum increase in root length (36%) was recorded by *Weissella* sp. treated seedlings as compared to the control whereas the shoot length recorded was 99% significantly higher in *Lactobacillus* sp. treated seedlings as compared to the control (Table 4). The treatment *Weissella* sp. resulted in 40% higher root fresh weight as compared to the control (Table 4). Root dry weight of the *Weissella sp.* treated seedlings was 13% higher compared to control (Table 4) and 32% as compared to *Lactobacillus* sp. The treatment with *Lactobacillus* sp. resulted in significant increase (107%) in shoot fresh weight as compared to the control (Table 4). Dry weight of shoot of the *Weissella sp.* treated seedlings was notably increased which was 21% as compared to the control (Table 4). The number of lateral and sub-lateral roots were recorded maximum (107% and 169%) by *Lactobacillus* sp. treated seedlings respectively as compared to control and *Weissella sp* (Table 4).

**Table 4:** Effect of *Lactobacillus* and *Weissella* spp. on root length, shoot length, root fresh weight, root dry weight, shoot fresh weight, shoot dry weight, no. of lateral roots and no. of sub-lateral roots of basmati rice seedlings in petri plate experiment.

Treatments	Root length (cm)	Shoot length (cm)	Root fresh weight (g)	Root dry weight (g)	Shoot fresh weight (g)	Shoot dry weight (g)	No. of lateral roots	No. of sub-lateral roots
Control	4.63^ab^	5.15^c^	0.029^a^	0.012^ab^	0.016^c^	0.0034^c^	4.33^b^	13^b^
*Weissella* sp.	6.33^a^	7.66^b^	0.040^a^	0.014^a^	0.026^b^	0.0041^b^	7.33^a^	15^b^
*Lactobacillus* sp.	4^b^	10.25^a^	0.038^a^	0.011^b^	0.036^a^	0.0055^a^	9.00^a^	35^a^

All means, which are not sharing letter, are significantly different at *P* < 0.05 while those are sharing are non-significant. Experiment was performed in three replicates.

### Pot experiment

#### Scoring of sheath blight (*R. solani*) disease in basmati rice

The scoring of *R. solani* disease was done at seventh day of application of fungal stress. The scale for the scoring of the sheath blight disease was 0–5 as shown in the Table 5. The *R. solani* treatment showed 90–100% disease susceptibility and had all the symptoms of disease such as yellowing of leaves with damping and lesion. *R. solani* + *Fungicide* treatment showed 50–90% disease susceptibility against *R. solani* showing the symptoms of yellowing of one or two leaves with damping and lesions. *Lactobacillus* sp. + *R. solani* treatment showed 50% disease severity and were moderately resistant against *R. solani*. *Weissella* sp. + *R. solani* showed 70% disease severity and were moderately resistant to moderately susceptible against *R. solani.*

**Table 5:** Scale for the scoring of sheath blight caused by *Rhizoctonia solani* in pot experiment.

Scale	Score (%)	Susceptibility	Symptoms	Treatment
0	0	Resistant	No symptoms	No treatment showed 0% inhibition
1	10–30	Moderately Resistant	Only tips yellow	*Lactobacillus* sp. + *R. solani*, *Weissella* sp. + *R. solani*
2	30–50	Moderately Resistant	Partly yellow with one leave damping	*Lactobacillus* sp. + *R. solani*, *Weissella* sp. + *R. solani*
3	50–70	Moderately susceptible	One or two leaves fully yellow with damping	*Weissella* sp. + *R. solani*, Fungicide + *R. solani*
4	70–90	Susceptible	Yellowing with damping and one or two lesions	Fungicide + *R. solani*
5	90–100	Highly Susceptible	All symptoms	*R. solani*

### Growth parameters of basmati rice in pot experiment

#### Shoot and root length, shoot fresh and dry weight, root fresh and dry weight

**Figure 1:**
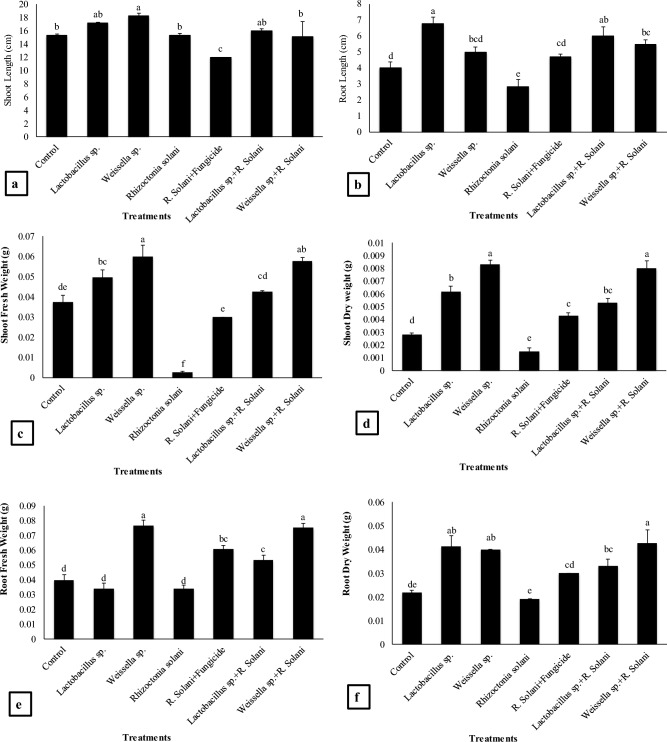
Effect of treatments on (**a**) shoot length, (**b**) root length, (**c**) shoot fresh weight, (**d**) shoot dry weight, (**e**) root fresh weight, (**f**) root dry weight of basmati rice. Data are expressed as mean ± standard deviation of three replicates. All means which are not sharing letter are significantly different at *P* < 0.05 while those are sharing are non-significant

In pot experiment, maximum increase in shoot length of Basmati rice was recorded by *Weissella* sp. treatment which resulted in 19% increase as compared to the normal control and *R. solani* (Fig. 1a). Similarly, *Lactobacillus* sp. alone showed non-significant increase (11%) in shoot length as compared to the control and under fungal stress (*Lactobacillus* sp. + *R. solani*) showed significant increase (5%) as compared to the *R. solani* treated plants. The minimum shoot length was recorded by *R. solani* + Fungicide treatment which was 22% decrease as compared to *R. solani* and normal control. A 12% increase in shoot length was recorded by *Lactobacillus* sp. over *R. solani* treatment.

A remarkable increase in root length (138% and 76%) was observed in *Lactobacillus* sp. and *Weissella* sp. treatments respectively as compared to the *R. solani* and 19% and 12% as compared to control respectively (Fig. 1b). The *Lactobacillus* sp. + *R. solani* treatment resulted in 112% increase in root length while the *Weissella* sp. + *R. solani* treatment showed 92% increase in root length as compared to the *R. solani* respectively.

The *Weissella* sp. + *R. solani* treatment resulted in maximum increase (54%) in shoot fresh weight as compared to the control and 2094% as compared to the *R. solani* (Fig. 1c). The fresh weight of shoot was significantly high in *Weissella* sp. + *R. solani* treatment (60%) as compared to the control and 2183% as compared to the *R. solani*. The *Lactobacillus* sp. treatment showed 34% increase in shoot fresh weight as compared to the control and 1794% as compared to the *R. solani*. Similarly, the *Lactobacillus* sp. + *R. solani* treatment resulted to increase shoot fresh weight by 14% as compared to the control and 1524% significant increase in weight was observed as compared to the *R. solani*.

Shoot dry weight was remarkably higher in *Weissella* sp. treatment (197%) as compared to the control (Fig. 1d). The treatment *Lactobacillus* sp. showed 121% increase in shoot dry weight as compared to the control and 314% as compared to the *R. solani*. The *Lactobacillus* sp. + *R. solani* treatment showed 91% increase in shoot dry weight as compared to the *R. solani* and 256% as compared to the *R. solani*. The 186% increase in weight was recorded by *Weissella* sp. + *R. solani* treatment as compared to the control and 434% compared to the *R. solani*. The *R. solani* + Fungicide treatment showed 54% increase in dry weight as compared to the control and 186% as compared to the *R. solani*.

Maximum increase in root fresh weight was recorded by *Weissella* sp. alone and in combination with fungal stress. The amount of increase in root fresh weight was 90% by *Weissella* sp. + *R. solani* treatment as compared to the control and 122% as compared to the *R. solani* (Fig. 1e). The *Lactobacillus* sp. + *R. solani* treatment resulted in 35% significant increase in root fresh weight as compared to the control and 58% as compared to *R. solani*. The *Weissella* sp. treatment showed 93% increase in root fresh weight as compared to the control and 126% as compared to the *R. solani*. The maximum root dry weight (95% and 90% respectively) was recorded by the *Weissella* sp. + *R. solani* and then by *Lactobacillus* sp. treatment as compared to the control (Fig. 1f). Root dry weight of *Weissella* sp. treatment was 83.4% significantly higher as compared to the control and 108% as compared to the *R. solani*. The treatment *Lactobacillus* sp. + *R. solani* resulted in 52% increase in root dry weight as compared to the control and 71% as compared to the *R. solani*. The *R. solani* + Fungicide treatment showed 37% increase root dry weight as compared to the control and 56% as compared to the *R. solani*.

#### Plant fresh and dry weight, number of lateral and sub-lateral roots

**Figure 2:**
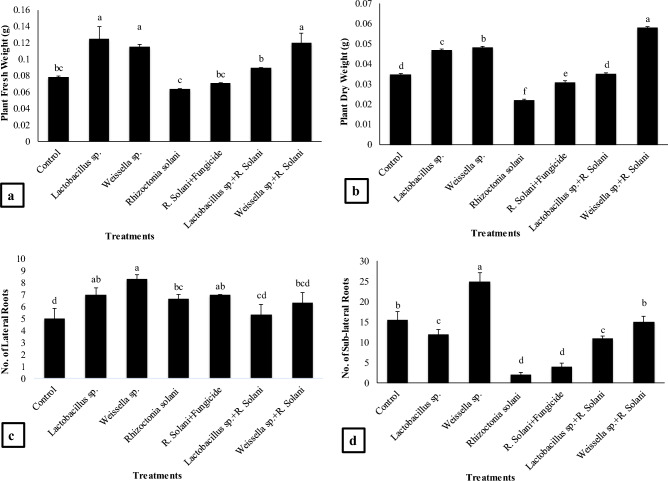
Effect of treatments on (**a**) plant fresh weight, (**b**) plant dry weight, (**c**) no. of lateral roots and (**d**) no. of sub-lateral roots. Data are expressed as mean ± standard deviation of three replicates. All means which are not sharing letter are significantly different at *P* < 0.05 while those are sharing are non-significant

The *Lactobacillus* sp. treatment resulted in the maximum (59%) increase in plant fresh weight as compared to the control and 94% increase as compared to the *R. solani* (Fig. 2a). The *Weissella* sp. treatment showed 46% rise in plant weight as compared to the control and 78% as compared to the *R. solani*. The *Lactobacillus* sp. + *R. solani* treatment showed 40% as compared to the *R. solani* while treatment *Weissella* sp. + *R. solani* resulted in 86% increase as compared to the control and *R. solani*.

The *Weissella* sp. + *R. solani* treatment resulted in maximum increase (67%) in plant dry weight as compared to the control and 163% as compared to the *R. solani* (Fig. 2b). The *Weissella* sp. treatment showed 40% increase as compared to the control and 120% as compared to the *R. solani*, moreover, the *Lactobacillus* sp. treatment showed 34% increase as compared to the control and 113% increase in plant dry weight as compared to the *R. solani* treatment.

The no. of lateral roots was significantly high in *Weissella* sp. treatment (66%) alone as compared to the control and 25% as compared to the *R. solani* (Fig. 2c). The *Lactobacillus* sp. treatment showed 40% increase in number of lateral roots as compared to the control and 5% as compared to the *R. solani*. The *Lactobacillus* sp. treatment showed 6% significant increase in number of lateral roots as compared to the control and 20% decrease as compared to the *R. solani*.

The remarkable increase (61%) in the number of sub-lateral roots was shown by *Weissella* sp. treatment as compared to the control and 1150% as compared to the *R. solani* (Fig. 2d). The *Lactobacillus* sp. treatment showed 500% increase in no of sub-lateral roots as compared to the *R. solani*. The *R. solani* + Fungicide treatment showed 100% increase compared to the *R. solani*. The *Lactobacillus* sp. + *R. solani* treatment showed 450% increase in the number of sub-lateral roots as compared to the *R. solani* while *Weissella* sp. + *R. solani* resulted in 650% increase as compared to the *R. solani* treatments.

### Photosynthetic pigments and carotenoid content

**Figure 3:**
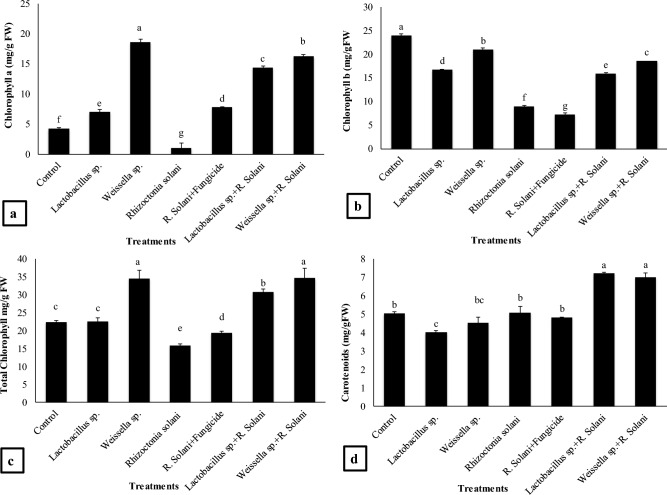
Effect of treatments on (**a**) chlorophyll a, (**b**) chlorophyll b, (**c**) total chlorophyll and (**d**) carotenoids content of basmati rice in pot experiment. Data are expressed as mean ± standard deviation of three replicates. All means which are not sharing letter are significantly different at *P* < 0.05 while those are sharing are non-significant

Application of *Weissella* sp. resulted in maximum increase of 332% in chlorophyll a content (Fig. 3a) as compared to the control and 1784% increase was recorded as compared to the *R. solani* followed by the treatment *Weissella* sp. + *R. solani* that showed 276% increase in chlorophyll a as compared to the control and 1541% as compared to the *R. solani*. *Lactobacillus* sp. also showed significant increase as compared to the control while *Lactobacillus* sp. + *R. solani* showed 105% increase over *R. solani* treatment. The chlorophyll b content in *Weissella* sp. treatment showed 134% rise as compared to the *R. solani* (Fig. 3b). The *Lactobacillus* sp. treatment resulted in 87% increase while *Lactobacillus* sp. + *R. solani* treatment showed 77% increase as compared to the *R. solani*. The Maximum increase of 107% in chlorophyll b content was exhibited by *Weissella* sp. + *R. solani* treatment as compared to the *R. solani*. *Weissella* sp. + *R. solani* treatment exhibited maximum total chlorophyll content (54%) over the control and 118% as compared to the *R. solani* (Fig. 3c). The *Lactobacillus* sp. + *R. solani* treatment showed significant increase (37%) as compared to the control and 94% as compared to the *R. solani*.

Maximum Carotenoid content was shown by *Lactobacillus* sp. + *R. solani* (42%) as compared to control and 41% as compared to *R. solani* (Fig. 3d). Treatment *Weissella* sp. + *R. solani* resulted in 38% increase in carotenoid content as compared to control and 37% as compared to *R. solani*.

### Proline, protein content, total soluble sugars, catalase (CAT), ascorbate peroxidase (APX), peroxidase (POD) activity

**Figure 4:**
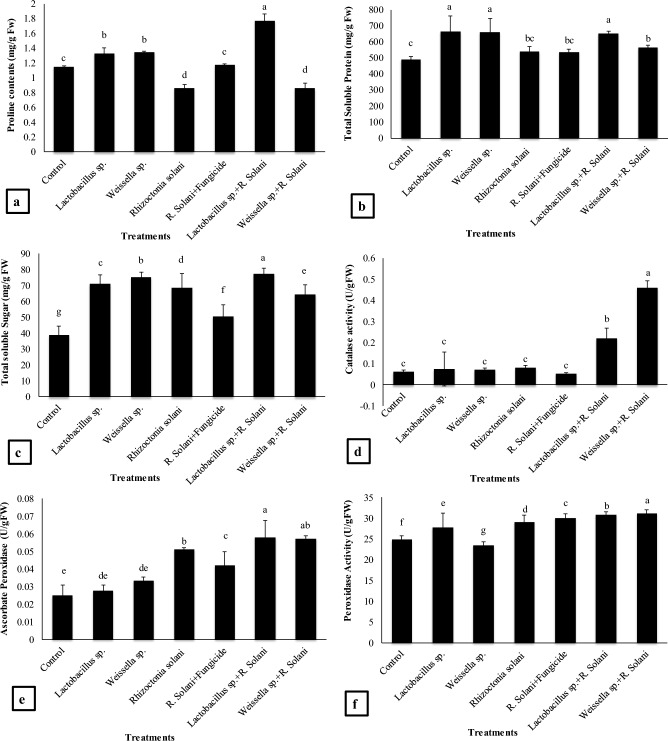
Effect of treatments on (**a**) proline, (**b**) total soluble protein content, (**c**) total soluble sugars content, (**d**) catalase activity (CAT), (**e**) ascorbate peroxidase activity (APX) and (**f**) peroxidase activity (POD) of basmati rice in pot experiment. Data are expressed as mean ± standard deviation of three replicates. All means which are not sharing letter are significantly different at *P* < 0.05 while those are sharing are non-significant

Results in Fig. 4a indicated that maximum proline content (54%) was recorded by *Lactobacillus* sp. + *R. solani* treatment as compared to the control and 106% as compared to the *R. solani* treatment. The *Lactobacillus* sp. alone treatment resulted in 15% increase as compared to the control and 54% as compared to the *R. solani*. A 17% increase in proline contents were recorded by *Weissella* sp. as compared to the control and 56% as compared to the *R. solani*. The combined treatment *R. solani* + Fungicide resulted in significant increase in proline contents as compared to *R. solani* treatment alone.

Significant increase in protein content was found in *Lactobacillus* sp. (35%) treatment over the control and 22% as compared to the *R. solani* (Fig. 4b). The *Weissella* sp. treatment showed 34% increase as compared to the control and 21% as compared to the *R. solani*. The *Lactobacillus* sp. + *R. solani* treatment resulted in 33% increase as compared to the control and 20% as compared to the *R. solani* while *Weissella* sp. + *R. solani* treatment resulted in 15% increase in protein content as compared to the control and showed non-significant increase as compared to the *R. solani* treatment.

Treatment *Lactobacillus* sp. + *R. solani* resulted in maximum increase in total soluble sugar content (100%) as compared to the control and 13% as compared to the *R. solani* (Fig. 4c) followed by *Weissella* sp. + *R. solani* which resulted in 66% increase in sugar content as compared to the control. The *Weissella* sp. treatment exhibited 94% while *Lactobacillus* sp. resulted in 83% significant increase in sugar content as compared to the control.

*Weissella* sp. + *R. solani* resulted in maximum increase in catalase activity by 650% and 458% as compared to control and *R. solani* treatments respectively. The *Lactobacillus* sp. + *R. solani* had been found to increase catalase activity by 261% as compared to the control and 167% increase as compared to the *R. solani*. Treatments *Lactobacillus* sp. and *Weissella* sp. resulted in 16% and 23% increase in catalase activity respectively compared to control (Fig. 4d).

Figure 4e showed that ascorbate peroxidase (APX) activity was increased by all treatments however; maximum significant increase (131% and 13%) was recorded by *Lactobacillus* sp. + *R. solani* treatment as compared to control and *R. solani* treatment respectively. *Weissella* sp. + *R. solani* increased 128% APX activity as compared to control and 12% as compared to the *R. solani* whereas *Lactobacillus* sp. and *Weissella* sp. applications resulted in 10% and 33% increase in APX activity respectively as compared to the control.

Maximum significant increase in the peroxidase (POD) activity was shown by *Weissella* sp. + *R. solani* treatment as compared to the control and all other treatments. The value of increase by the respective treatments was 25%, 7% and 32% as compared to control, *R. solani and Weissella* sp. alone. The *Lactobacillus* sp. + *R. solani* application had also been found to show the maximum increase by 25% in POD activity as compared to the control and 6% as compared to the *R. solani* while *Lactobacillus* sp. single applications resulted in 11% increase as compared to the control (Fig. 4f).

### Superoxide dismutase (SOD), polyphenyl oxidase (PPO) and phenyl-alanine ammonia lyase (PAL) activity

**Figure 5:**
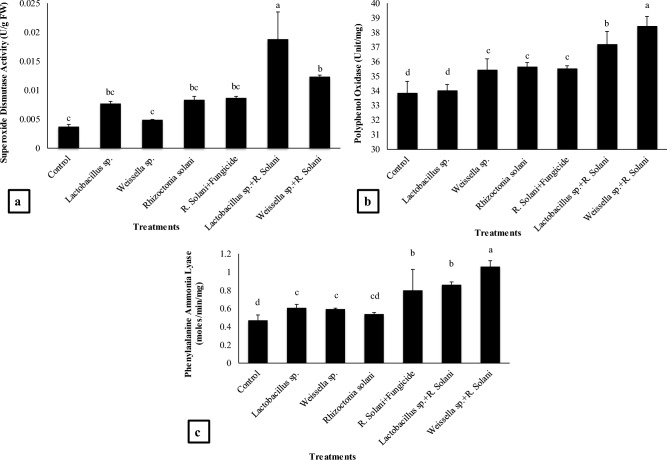
Effect of treatments on (**a**) superoxide dismutase (SOD) (**b**) polyphenol oxidase, and (**c**) phenylalanine ammonia lyase activity of basmati rice in pot experiment. Data are expressed as mean ± standard deviation of three replicates. All means which are not sharing letter are significantly different at *P* < 0.05 while those are sharing are non-significant

Figure 5a showed that significant increase of 400% in SOD activity was recorded by *Lactobacillus* sp. + *R. solani* treatment followed by 227% by *Weissella* sp. + *R. solani* treatment over the control. Application of *Lactobacillus* sp. under stress of *R. solani* resulted in 125% increase in SOD activity as compared to *R. solani* treatment. Moreover, *Lactobacillus* sp. alone resulted in 104% increase as compared to *R. solani* and 30% increase as compared to control. Similarly, *Weissella* sp. + *R. solani* treatment resulted in 47% increase in SOD activity as compared to *R. solani* treatment.

Maximum increase in polyphenol oxidase (PPO) activity was recorded by *Weissella* sp. + *R. solani* treatment as compared to control and all other treatments. The amount of increase by the respective treatment was 14%, 7% and 8% as compared to the control, *R. solani,* and *Weissella* sp. alone. The *Lactobacillus* sp. + *R. solani* treatment resulted in 10% increase in PPO activity as compared to control and 4% increase as compared to the *R. solani* whereas *Weissella* sp. alone increased 4% PPO activity as compared to the control (Fig. 5b).

The results presented in Fig. 5c indicated the increase in phenylalanine ammonia lyase (PAL) activity in all the treatments as compared to the control and fungal pathogen. The maximum increase (124%, 95%, 79%) in PAL activity was recorded by *Weissella* sp. + *R. solani* as compared to the control, *R. solani* and *Weissella* sp. respectively. The treatment *Lactobacillus* sp. + *R. solani* also significantly increased PAL activity by 82% as compared to control and 58% as compared to the *R. solani* while *Lactobacillus* sp. and *Weissella* sp. single applications resulted in 29% and 25% increase in PAL activity respectively as compared to the control and 12% and 9% as compared to the *R. solani* ().

## Discussion

In the present study, fungicidal effect of two biocontrol agents *Lactobacillus* sp. and *Weissella* sp. was studied against *R. solani,* causing sheath blight disease in basmati rice. Sheath blight is the most devastating disease of rice crop and decreases the crop productivity by almost 45%^16^. It was observed that both strains exhibited the antifungal activity against *R. solani*. However, *Lactobacillus* sp. resulted in maximum percentage inhibition (81%) while *Weissella* sp. exhibited 60% inhibition against of *R. solani*. Bazukyan et al.^17^ reported the antifungal activity of *Lactobacillus* sp. against *Penicillium aurantioviolaceum* and *Mucor plumbeus* which is in consistence with the current study*.* The inhibitory effect might be due to the production of different chemical substances or metabolites by the bacteria which might inhibit the growth of the fungus. *Lactobacillus* sp. has been reported to produce secondary metabolites like organic acid, bacteriocins, aflatoxin and hydrogen peroxide that acted as an antifungal agent against *Aspergillus flavis*^18^.

Baek et al.^19^ reported the antifungal activity of *Weissella* sp. on other fungal species including *Cladosporium sp*. and *Neurospora* sp. in rice cake which are in line with our findings. The reason for the inhibition of *R. solani* by *Lactobacillus* sp. and *Weissella* sp. might be that both the bacterial strains are lactic acid bacteria (LAB) and LAB produce organic acids mainly lactic acid and acetic acid that have been reported to decrease fungal activity of other fungi and related pathogens^19^.

In the next step, we evaluated the germination percentage of the Basmati rice inoculated with *R. solani* followed by the applications of biocontrol agents*. Lactobacillus* sp. treated seeds resulted in 100% germination percentage as compared to normal control (93.3%). Similar findings have been reported by Suman et al.^20^ where *Pseudomonas flourescens* inoculation resulted in 100% germination as compared to normal control (90%) in rice against *R. solani* causing sheath blight disease.

*Lactobacillus* sp. exhibited 10–50% and *Weissella* sp. resulted in 10–70% resistance to *R. solani* stress as compared to fungal control without inoculation. Similarly, França et al.^21^ demonstrated the inhibition of sheath blight disease caused by *R. solani* in rice plants using *Trichoderma asperellum* as a biocontrol agent, the disease severity was decreased about 26% and crop yield was enhanced by 41%. *Bacillus subtilis* and *megaterium* as biocontrol agents have been reported to inhibit the sheath blight caused by *R. solani* in rice by 60.20% and 98.5% respectively^22,23^. The increased resistance provided by *Lactobacillus* sp. and *Weissella* sp. might be due to the fact that these strains were reported to produce antifungal compounds (organic acid, hydrogen peroxide) that help to reduce the fungal stress in rice plants. Moreover, in current study, *Weissella* sp. resulted in siderophore production (1.46 ± 0.03) and have also been found to solubilize phosphate (4.45 ± 0.65) which led to the enhanced plant weight and root and shoot length. *Lactobacillus* sp. also shown to produce siderophore and solubilize phosphate about 144% and 52% respectively.

In the current study, maximum root length was recorded by *Lactobacillus* sp. by 69% as compared to control and by 138% as compared to *R. solani* followed by *Weissella* sp. while maximum shoot length was exhibited by *Weissella* sp. as compared to control and *R. solani*. The results are in agreement with Verma et al.^24^ where they demonstrated that in the presence of biocontrol agent, *Pantoea hericii*, the rice seedlings showed maximum root and shoot length as compared to the normal control. Similar findings have been demonstrated by Selim et al.^25^. The increase in root and shoot length in *Lactobacillus* sp. and *Weissella* sp. could be because of the reported ability of biocontrol agent to produce growth promoting hormones or chemicals that increase the root length.

*Lactobacillus* sp. resulted in maximum plant fresh weight while *Weissella* sp. showed maximum plant dry weight as compared to control. These results are in consistent with findings by Srivastava et al.^26^ who reported the similar results that biocontrol agent *Bacillus amyloliquefaciens* increased the fresh and dry weight of rice plant as compared to rice plants which were inoculated with *R. solani* infected with sheath blight disease. The reason of the increase in weight in the probiotic treated rice plants might be that the probiotics were reported to release hormones that increase the mass and vigor of the plant and increase the plant weight^18^.

Maximum chlorophyll (a and b) was shown by *Weissella* sp. while total chlorophyll and carotenoids content were maximum in rice plants treated with *Weissella* sp. and *Weissella* sp. + *R. solani* respectively. Doni et al.^27^ reported the increased chlorophyll content of rice seedlings treated with *Trichoderma* biofertilizer as compared to control against *R. solani* and these results are in line with our findings. Srivastava et al.^26^ and Suman et al.^20^ also reported the similar findings. The increase in the chlorophyll content in bacterial inoculated plants could be due to the reason that these strains enhance the health, vigor and carbon assimilation of the basmati rice. The fungal inoculation resulted in reduction in chlorophyll because the *R. solani* infection in basmati rice causes cell wall loosening and desertification of pectin^28^.

In the current study, *Lactobacillus* sp. + *R. solani* application exhibited maximum proline content compared to control and *R. solani*. These findings are in accordance with the results by Hashem et al.^29^ where they reported the increased proline content in mung bean inoculated with *Bacillus subtilis* and infected with *Macrophomina phaseolina* as compared to the control*.* Moreover, *Lactobacillus* sp. + *R. solani* gave maximum level of sugar contents by 100% as compared to control. Similar increase in sugar content of the rice seedling has been observed by Srivastava et al.^26^ following treatment with biocontrol bacteria and *Rhizoctonia solani* pathogen as compared to the normal control. It has been observed that under stress conditions, plant adjusts its metabolism by accumulating various osmolytes or solutes. These osmolytes or solutes mainly include sugars, proline and other polyamines. The biocontrol agent or probiotics are reported to increase the proline and sugar content under biotic or abiotic stresses which then reduce stress and improve plant growth^30^.

To expand our knowledge, we also analyzed the defense enzymes catalase, SOD, POD, PAL, PPO and APX in the current study and it was observed that the activities of all the defense related enzymes were significantly increased. Maximum SOD activity was recorded by *Lactobacillus* sp. + *R. solani* as compared to *R. solani*. Nassimi et al.^31^ reported the elevated level of SOD activity in the treatment of biological control agent + *R. solani* as compared to the control and the bacterial control treatment. The SOD is the first line defense of antioxidant enzymes and acts by scavenging oxidative radicals^32^. In the current study, maximum SOD activity in *Weissella* sp. + *R. solani* was due to the activation of defense mechanism in fungus inoculated plants which ultimately improved plant growth.

Treatment *Lactobacillus* sp. + *R. solani* increased CAT activity but maximum activity was recorded by *Weissella* sp. + *R. solani* as compared to control and *R. solani* and these results are in line with the findings of Srivastava et al.^26^ where they found the increased catalase activity in rice plants inoculated with *Bacillus amyloliguefaciens* alone and also the rice seedlings infected with *Rhizoctonia solani* + *Bacillus amyloliguefaciens*. Shamim et al.^33^ also reported the similar findings. When plant is exposed to biotic and abiotic stress, it leads to the production of reactive oxygen species (ROS) which in turn causes oxidative damage to the plant and alter the plant metabolism and normal functions. To adjust the enhanced production of ROS species, plant induces antioxidant defense system which includes production of enzymes called catalase (CAT), superoxide dismutase (SOD), ascorbate peroxidase (APX), polyphenol oxidase (PPO) and phenyl-alanine ammonia lyase (PAL)^34^. The CAT, APX, POD, PPO and PAL are activated as a second line of defense enzyme system and plant increases their production by the application of biocontrol agent because biocontrol agents are reported to alter antioxidant enzyme activity when applied to plant under stress conditions^35^.

Maximum POD was resulted by *Weissella* sp. + *R. solani* and similar results were recorded by Shabanamol et al.^36^ who reported the increased POD activity in rice plants inoculated with *Lysinibacillus sphaericus* which was infected with *R. solani* against sheath blight. The POD also acts as second line defense and increase in the POD in the combine treatment of probiotic control treatment resulted in improved plant growth. Almost all the applications gave an increase in APX activity, but the maximum activity was shown by *Lactobacillus* sp. + *R. solani* application as compared to control and *R. solani*. Hashem et al.^29^ reported the similar results where APX activity was increased by 24.63% following the treatment of bacterial agent under fungal pathogen induced stress as compared to the control.

Treatment *Weissella* sp. + *R. solani* has been found to give maximum PPO activity as compared to *R. solani*. Similar results have been demonstrated by Rais et al.^37^ where they reported an increased amount of PPO in the super basmati rice inoculated with *Bacillus spp*. and infected with *Pyricularia oryzae* as compared to the *Bacillus spp*. and also observed improvement in plant growth.

Other applications also resulted in increased PAL activity but *Weissella* sp. + *R. solani* exhibited maximum activity as compared to control. Shamim et al.^33^ reported the increase in PAL activity in rice varieties infected with *Rhizoctonia solani* which are in agreement with our results. Akladious et al.^38^ also reported the increased PAL activity in faba bean inoculated with biocontrol surfactant isolated from *Bacillus subtilis* and infected with *R. solani.*

## Conclusion

Present study infers that *Lactobacillus* sp. exhibited maximum inhibition of growth of *R. solani*. The germination percentage of rice seedlings treated with *Lactobacillus* sp. showed the maximum growth. The treatment *Lactobacillus* sp. and *Weissella* sp. both resulted in maximum increase in fresh and dry weight of Basmati rice plant. Treatment of *Lactobacillus* sp. + *R. solani* exhibited increased proline and sugar contents. The elevated levels of antioxidant enzymes SOD, POD, CAT, PPO and PAL have been found in both *Weissella* sp. + *R. solani* and *Lactobacillus* sp. + *R. solani* treatments. The *L. rhamnosus* showed enhanced antifungal activity against *R. solani* and *Weissella* sp. showed enhanced protection of basmati rice against *R. solani* thus these bacteria can be suggested as potential biocontrol agent.

## Materials and methods

*Lactobacillus* sp. (accession no. OR100385) and *Weissella* sp. (accession no. OR100388) were isolated and purified from seeds of melon and were used as an antifungal agent against pathogenic fungi *Rhizoctonia solani (R. solani).* Melon seeds were obtained from the melon fruit which was purchased from local market.

Furthermore, the plant material collected and used in this study completely complies with institutional, national, and international guidelines and legislation regarding this type of experiment.

### Determination of antifungal potential of *Lactobacillus* and *Weissella* spp.

Biocontrol potential of *Lactobacillus* and *Weissella* sp. was evaluated as an antifungal agent by the method of Wahyudi et al.^39^. Both microbes were inoculated in nutrient broth and then 70 µL of broth culture was taken and spread on the potato dextrose agar uniformly. Then *R. solani* was inoculated in the center of the medium and incubated at 28°C. Growth inhibition of *R. solani* was recorded after seven days of inoculation.

### Phosphate solubilization, hydrogen cyanide and siderophore production assays of *Lactobacillus* and *W. confusa spp*.

Plant growth promoting potential of *Lactobacillus*. and *Weissella* spp.was evaluated by performing phosphate solubilization, hydrogen cyanide and siderophore production assays. Phosphate solubilization assay was performed using the method proposed by Setia and Mutmainnah^40^. One-day old culture of *Lactobacillus* sp. and *Weissella* sp. were placed in the center of the prepared Pikovskaya (PVK) medium plates by spot inoculation method. Plates were incubated at 37°C for seven days and formation of halozone was recorded.

Lorck^41^ method was used for the screening of hydrogen cyanide (HCN) production. Nutrient agar mixed with glycine (4.4 g/L) was prepared and poured in petri plates. Whatman filter paper was soaked in 2% sodium carbonate and 0.5% picric acid and was placed at the inner surface of the lid of petri plate. The bacteria were then streaked on agar plates and incubated at 30°C for four days. Appearance of orange to red color indicated the production of HCN.

Schwyn and Neilands^42^ Chrome azurol sulphonate (CAS) assay was used for the detection of siderophore. Two types of media were prepared. One media consisted of Chrome azurol S, hexadecyltrimethyl ammonium bromide (HDTMA) and second was King’s media. The two media were mixed and autoclaved. At the time of pouring, 1 mM FeCl_3_.6H_2_O amended with 300 µL HCl was added in media. One day old colonies were picked and placed at the center of the plate by spot inoculation method. Plates were incubated for seven days. The appearance of blue to yellow zone indicated the siderophore production.

### Growth promoting potential of *Lactobacillus* and *Weissella* spp. of basmati rice in petri plate experiment

For the determination of effect of *Lactobacillus* and *Weissella* spp. on seed germination and growth promotion of Basmati rice, a petri plate experiment was performed (Supplementary Fig. 3) in completely randomized design (CRD) with three replications. Basmati rice seeds were collected from Plant Genetic Resources Program (National Agricultural Research Centre). Then surface sterilization of seeds was carried out with 70% ethanol for 2 min with continuous shaking and then seeds were washed with 3–5 times with distilled water. *Lactobacillus* and *Weissella* spp. were inoculated and seeds were placed in petri plates containing autoclaved/sterilized cotton and filter paper. Germination data was recorded until all the seeds were germinated.

To observe the effect of both microbes on the basmati rice, root length, shoot length, root fresh weight, root dry weight, shoot fresh weight, shoot dry weight, number of lateral roots and number of sub-lateral roots was recorded after harvesting. The treatments applied on Basmati rice in petri plate experiment in vitro were control, *Lactobacillus* sp. and *Weissella* sp..

### Pot experiment

A pot experiment was conducted at the Department of Biosciences, COMSATS University Islamabad in order to determine the biocontrol potential of *Lactobacillus* and *Weissella* spp. against *R. solani* on Basmati rice (Supplementary Fig. 4).

Basmati rice seeds were first grown into rice seedlings in ceramic plates. These seedlings (10) were planted into pots after inoculation with *Lactobacillus* sp. and *Weissella* sp. Seven treatments applied on Basmati rice in pot experiment; Control, *Lactobacillus* sp., *Weissella* sp., *R. solani* , Fungicide + *R. solani*, *Lactobacillus* sp. + *R. solani* and *Weissella* sp. + *R. solani*. In fungicide control treatment, 62% chlorothalonil fungicide was added. *R. solani* was inoculated at the third leaf stage of Basmati rice. Canopy was made around the pots and covered with plastic sheet to maintain humidity for 2–3 days. Daily observations were made, and incidence of disease was evaluated from 4 to 7th day. Three symptoms were mainly recorded, yellowing, damping off and lesions formation at the scoring scale of 0–5. All the plants were harvested and stored at 4°C for further biochemical test.

### Growth parameters

At the time of harvesting, root length (RL), shoot length (SL), root fresh weight (RFW), shoot fresh weight (SFW) were measured. After drying the samples in oven at 70°C for 72 h, dry weight was recorded.

### Measurement of photosynthetic pigments, carotenoid, proline, protein content and total soluble sugars (TSS),

Photosynthetic pigments (Chlorophyll a and b) and carotenoid content was determined following the method of Hiscox and Israelstan^44^ with some modifications. Fresh tissue (0.01 g) from each treatment was soaked in 1 mL of dimethyl sulfoxide (DMSO) in the test tubes. These samples were then incubated at 65°C for 60 min. After incubation, the absorbance was measured using spectrophotometer at 663, 645 and 470 nm for chlorophyll a, b and carotenoids respectively.

Proline content was measured using the method described by Carillo and Gibon^46^. Grinding of 0.1 g of fresh leaves was carried out in 1 mL of 80% ethanol followed by centrifugation at 4000 rpm for 15 min, then supernatant were transferred to the test tubes. Two flasks were taken. In one flask, 20 mL of distilled water and 30 mL of acetic acid were added. Then 10 mL of phenol and 20 mL of distilled water were taken in the other flask. The two solutions were mixed and raised the volume upto 100 mL and then ninhydrin was added. Reaction mixture (1000 µL) was added in 500 µL of crude enzyme extract and absorbance was measured at 520 nm.

Lowry et al.^45^ method was used to determine the protein content of leaves. Grinding of the fresh leaves (0.1 g) tissue was carried out in 1 mL of phosphate buffer of pH 7.5, after that, sample was centrifuged for 10 min at 3000 rpm. Supernatant (0.1 mL) was transferred to the test tube and raised the volume upto 1 mL by adding distilled water. Copper sulphate (1000 µL) reagent and Folin-phenol reagent (100 µL) were added in the test tubes containing supernatant. Then absorbance was measured at 650 nm using spectrophotometer. Bovin serum albumin was used as a standard.

Total soluble sugars were determined using the method of Dubois et al.^43^. Fresh leaves (100 mg) were ground in 1 mL of distilled water and centrifuged at 4000 rpm for 15 min. Then 1 mL of 96% sulphuric acid and 0.3 mL of 5% phenol was added in the test tube containing supernatant. Incubation was carried out at room temperature and the absorbance was measured at 485 nm.

### Measurement of catalase (CAT), peroxidase (POD), Superoxide dismutase (SOD), Ascorbate peroxidase (APX), polyphenol oxidase (PPO) and Phenylalanine ammonia lyase (PAL) Activity

Catalase (CAT) activity was determined following the method of Kumar et al.^47^. Fresh leaves (0.5 g) were ground in 1 mL of sodium phosphate buffer (pH 7.0, 1% PVP) followed by centrifugation at 4000 rpm for 10–15 min. To 200 µL of crude extract, 500 µL of 0.5 M phosphate buffer (pH 7.0) and 100 µL of 3% hydrogen peroxide was added. Hydrogen peroxide (H_2_O_2_) was added in the reaction mixture just before the absorbance was measured at 240 nm for 3 min.

Ascorbate peroxidase (APX) was measured according to Nakano and Asada^50^. To 100 µL of crude extract, 600 µL of 50 mM sodium phosphate buffer (pH 7), 100 µL of 1 mM EDTA, 100 µL of 5 mM Ascorbate and 100 µL of H_2_O_2_ was added. In blank tube, 100 µL of phosphate buffer was added instead of enzyme sample. Then absorbance was measured at 290 nm for 3 min using spectrophotometer.

Zia et al*.*^48^ method was used to determine peroxidase (POD) activity. Reaction mixture was prepared by adding 1000 µL of 0.05 M Pyrogallol, 600 µL of 1% H_2_O_2_, and 100 µL of enzyme crude extract. Absorbance was measured at 420 nm for 3 min.

Superoxide dismutase (SOD) activity was determined by the method of Beauchamp and Fridovich^49^. Reaction mixture contained 2 mL of solution A, in 100 mL of phosphate buffer (pH 7.8) and the volume was increased upto 250 mL with phosphate buffer (pH 7.8), 0.5 mL of solution B (added 0.00113 g Riboflavin in 100 mL of phosphate buffer (pH 7.8). Then 40 mL of solution was taken, and the volume was raised upto 100 mL by adding 60 mL of phosphate buffer and 0.5 mL of crude extract. Two sets of reaction mixture were prepared. One was kept in dark and other set was kept in light at 30°C for 20 min. Then absorbance was taken at 560 nm.

Kara and Mishra^51^ method was used to describe polyphenol oxidase (PPO) activity with some modifications. Reaction mixture was prepared by adding 1.2 mL of 25 mM phosphate buffer pH 6.8, 250 µL of 0.1 M pyrogallol and 50 µL of enzyme extract. Absorbance was measured at 420 nm using pyrogallol as a blank.

Phenylalanine ammonia lyase (PAL) activity was determined using the method of Singh and Jha^52^. Fresh tissue (0.1 g) was ground in 1 mL of sodium borate buffer (pH 7.8) and was centrifuged at 3000 rpm for 10 min. Reaction mixture was prepared by adding 30 µL of enzyme extract, 670 µL of L-phenylalanine (3 mM in Tris–HCL buffer pH 8.5). For blank, 0.15 M Tris–HCl buffer was added in place of crude enzyme extract. Absorbance was taken at 290 nm.

### Statistical analysis

Statistical analysis of the data was done by Analysis of Variance (ANOVA) using Statistix software version 8.1. Steel and Torrie^53^ method was used to compare the mean values by least significant difference (LSD) at *P* < 0.05. Origin Pro 2016 (OriginLAB Northampton, MA) was used for the graphical representation of data.

### Supplementary Information

Supplementary Information 1.

Supplementary Information 2.

## Data Availability

All data generated or analysed during this study are included in this published article and its supplementary information files.
