# Supplementary material for: Biocontrol of Rhizoctonia solani in basmati rice by the application of Lactobacillus and Weissella spp
Source: Sci Rep. 2023 Aug 24;13:13855. doi: 10.1038/s41598-023-41058-9 (PMC10449839; doi:10.1038/s41598-023-41058-9)
Supplement: Supplementary file 2 — Supplementary Information 2. [file 41598_2023_41058_MOESM2_ESM.docx]

**Supplementary Figure 1 (A)** Antifungal activity of *Lactobacillus sp.* and *Weissella sp.* against *Rhizoctonia solani,* **(B)** Phosphate solubilization potential of *Lactobacillus sp.* and *Weissella sp.*

**Supplementary Figure 2 (A)** Siderophore production by *Lactobacillus sp.* and *Weissella sp.,* **(B)** HCN production by *Lactobacillus sp.* and *Weissella sp.*

**Supplementary Figure 3** Effect of *Lactobacillus* *sp.* and *Weissella sp* on Basmati Rice in petri plate experiment.


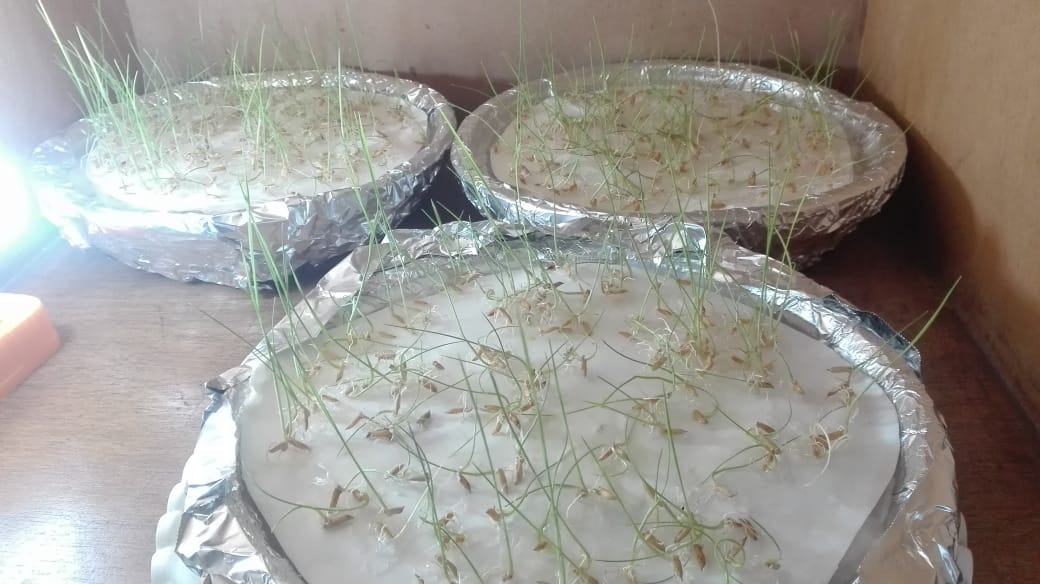


**Supplementary Figure 4** Pot Experiment on Basmati Rice using *Lactobacillus* *sp.* (MA) and *Weissella sp.* (MD) as biocontrol agent against *Rhizoctonia Solani*
